# A NEW METHOD TO IDENTIFY MEDICARE CLAIMS-BASED FACTORS ASSOCIATED WITH POST-ACUTE RECOVERY IN OLDER ADULTS WITH ADRD

**DOI:** 10.1093/geroni/igad104.3144

**Published:** 2023-12-21

**Authors:** Chixiang Chen, Biyi Shen, Jason Falvey, Michelle Shardell, Haoyu Ren

**Affiliations:** University of Maryland School of Medicine, Baltimore, Maryland, United States; Regeneron, Ellicott City, Maryland, United States; University of Maryland School of Medicine, Baltimore, Maryland, United States; University of Maryland School of Medicine, Baltimore, Maryland, United States; University of Maryland, Baltimore County, Baltimore, Maryland, United States

## Abstract

Nearly 300,000 older adults experience a hip fracture every year, the majority of which occur following a fall. Unfortunately, recovery from hip fracture is poor, where older adults diagnosed with Alzheimer’s Disease and Related Dementia (ADRD) spend a particularly long time in hospitals or rehabilitation facilities during the post-operative recuperation period. Because older adults value functional recovery and spending time at home versus facilities as key outcomes after hospitalization, identifying factors that influence days spent at home after hospitalization is imperative. However, few rigorous analytical approaches are available to help overcome potential sources of analysis bias such as hospital-level unmeasured confounders, informative hospital size, and loss to follow-up due to death. To overcome these challenges, we developed a new data science approach equipped with unsupervised learning to simultaneously handle statistical complexities that are often encountered in research using large administrative claims databases. The proposed approach is accessible and performs well in simulation studies. Analysis of 16562 Medicare beneficiaries diagnosed with ADRD who were aged 65 or older and experienced a hip fracture between 2017 and 2019 demonstrated that older age, longer length of stay in the hospital, more chronic health conditions, and less days at home before fracture are significantly associated less days spent at home in the 12-month period after hip fracture (p<.05), while existing methods fail to detect some effects. Findings demonstrate that the proposed method can overcome bias in health services research using Medicare data and can lead to findings that may inform care and resource allocation.

